# Diagnostic value of oral “beefy red” patch combined with fingertip blood mean corpuscular volume in vitamin B12 deficiency

**DOI:** 10.1186/s12903-022-02309-9

**Published:** 2022-07-05

**Authors:** Xiaoheng Xu, Yang Liu, Xiaoqin Xiong, Yanmei Yao, Huiting Hu, Xiao Jiang, Wenxia Meng

**Affiliations:** Departments of Oral Medicine, Stomatological Hospital, Southern Medical University, Guangzhou, 510260 Guangdong Province People’s Republic of China

**Keywords:** Vitamin B12 deficiency, Mean corpuscular volume, “Beefy red” patch, Diagnostic test, Diagnostic treatment

## Abstract

**Objectives:**

To investigate the diagnostic value of accessible fingertip mean corpuscular volume (MCV) combined with a visible “beefy red” patch in the diagnosis of vitamin B12 (VB12) deficiency in local clinics and hospitals without in-house clinical laboratories, especially in remote areas.

**Materials and methods:**

The medical history data of patients complaining of oral mucosal pain at the Stomatological Hospital of Southern Medical University were reviewed. All included patients underwent fingertip blood routine examination, specific serological test (serum VB12, folic acid, iron, and ferritin), and detailed oral clinical examinations. According to the results of the serum VB12 test patients were divided into case and control groups. In diagnostic test, the diagnostic value of the “beefy red” patch and elevated MCV in VB12 deficiency was evaluated by the receiver operator characteristic curve.

**Results:**

There were more female patients than male patients in the case group (serum VB12 level < 148 pmol/L, n = 81) and control group (serum VB12 level ≥ 148 pmol/L, n = 60), mostly middle-aged and elderly patients. There were no statistical differences in gender and age between the two groups. In the case group, the number of individuals with stomach disease was 13, the number of individuals with “beefy red” patch was 78, the number of individuals with oral ulcer was 29, the number of individuals with “MCV > 100fL” and “folic acid < 15.9 nmol/L” were respectively 68 and 5. All were more than that in control group (*P* < 0.05). The diagnostic test, “beefy red patch” has high sensitivity (0.963) but low specificity(0.883), “MCV > 100 fL” has high specificity (0.933) but low specificity (0.815), and “MCV > 100 fL combined with beefy red patch” has maximal specificity (0.950), and area under the curve (0.949).

**Conclusions:**

Visible oral “beefy red” patch combined with accessible fingertip blood MCV could improve the rate of diagnosis in VB12 deficiency, especially in the elderly in local clinics and hospitals without in-house clinical laboratories in China, which is conducive to early disease detection and treatment.

## Introduction

Vitamin B12 (VB12) deficiency, a nutritional deficiency disease caused by insufficient intake or malabsorption of vitamin B12, could cause a wide range of systemic diseases and oral lesions, resulting in devastating clinical and socioeconomic consequences [1]. The clinical manifestations of VB12 deficiency are diverse and non-specific. Systemic diseases include megaloblastic anemia [2], neurological symptoms [3], skin and mucous membrane pigmentation [1], and digestive system symptoms [4]. The oral manifestations include atrophic glossitis, recurrent aphthous ulcer (RAU) [5], burning mouth symptom, diffuse erythematous macules, opportunistic conditions as candidiasis, and angular cheilitis [6–8]. Due to the rapid renewal, the oral mucosal cells are susceptible to the effects of DNA synthesis disorders related to VB12 deficiency, and oral damage predates neurological symptoms [9–11]. Recently, some studies reported significant VB12 deficiency among symptomatic oral lichen planus patients [12]. It is challenging for oral medicine specialists to make an early diagnosis for VB12 deficiency.

The detection methods for VB12 deficiency include poor specific blood routine and blood smear examinations, traumatic bone marrow smears, deoxyuridine suppression experiments with radioactive contamination, time-consuming microbiological methods, expensive high-performance liquid chromatography, and urine methylmalonic acid determination [1, 13, 14]. Currently, the most commonly used method for clinical testing is serum VB12 determination [15]. The current guidelines for diagnosing VB12 deficiency recommend that the serum levels of the vitamin, folate, homocysteine, and methylmalonic acid be assessed concurrently due to their close correlation in metabolism [16]. However, the serum VB12 test is costly and time-consuming, requires venous blood, and is difficult to carry out in local clinics and hospitals without in-house clinical laboratories, especially the remote areas. Therefore, a rapid and convenient diagnosis of VB12 deficiency is clinically significant.

One of the clinical manifestations of VB12 deficiency is Hunter's glossitis, which gradually develops from diffuse bright red patches with mucosal pain (Beefy red patch), which can occur in any oral mucosa [17]. In VB12 deficiency, “beefy red patches” often accompanied by atrophic glossitis on oral mucosa were presumed to play a warning role [18]. Although these occur early, the red patches are often confused with other red lesions on oral mucosa, such as oral Candida infection [19, 20]. Oral medicine doctors should be cautious to prevent missed diagnosis and misdiagnosis, leading to delayed treatment. As we know, VB12 is involved in the production of bone marrow red blood cells. Thus, its deficiency will affect the division of red blood cells in the bone marrow, resulting in increased cell size and mean corpuscular volume (MCV). Thus, MCV > 100 fL contributes to the diagnosis of VB12 deficiency [21]. However, MCV can be normal or abnormal in the early stage when only the “Beefy red patches”appears [22]. Herein, we used a simple and fast detection method that combining “beefy red” patch and fingertip MCV to evaluate the diagnostic value for VB12 deficiency. This approach will bring benefits for the local clinics and hospitals without in-house clinical laboratories, especially the remote areas.

## Material and methods

### Subjects

The cross-sectional study was conducted by reviewing the cases Stomatological Hospital of Southern Medical University (Guangzhou, Guangdong, China) from April 2017 to October 2019. We consecutively selected patients with primary complaints of oral mucosal pain, burning sensation, and recurrent ulcers. Patients who met the following conditions were excluded: a history of cancer (except those who had undergone gastrectomy for stomach cancer), gout, and low serum potassium. All research subjects gave informed consent to this experiment, voluntarily cooperated and signed an informed consent form and the protocol was reviewed by the Institutional Ethics Committee of this hospital [approval no. (2019)13]. Finally, we recruited 141 patients. Sociodemographic information was collected (eg. name, age and gender) and examined their medical history and related laboratory tests. Also, the clinical manifestations and signs were evaluated and recorded by oral mucosal doctors with > 10 years of clinical experience.

Patients with erythematous candidiasis or geographic tongue were included into the control group. Patients with erythematous candidiasis often have a history of antibiotics or hormone administration. Diffuse fusion erythema is mainly located on the back of the tongue, showing red depressions or mucous membranes with dentures. These patients can be classified as Candida infection by taking samples of the exfoliated epithelium in the erythematous area for examination by smear method, and the lesions can be eliminated after antifungal treatment. Patients with geographic tongue present a ring shape, and irregular erythema appears on the front two-thirds of the back and the side of the tongue. In addition, a slight raised keratinization zone or line was noted around the lesion; the shape and location are changeable.

All included patients underwent fingertip blood routine examination, specific serological test (VB12, folic acid, iron, and ferritin), and detailed oral clinical examinations. The reference intervals of hemoglobin and MCV for adults were as follows: 11.0–15.0 g/dL in women, 13.0–17.0 g/dL in men, and 80–100 fL for MCV in all adults. The test items analyzed include serum and erythrocyte folate (15.9–72.5 nmol/L) and serum VB12 level (≥ 148 pmol/L). All tests cited above were performed in a single laboratory, with the same methods.

**Fig. 1:**
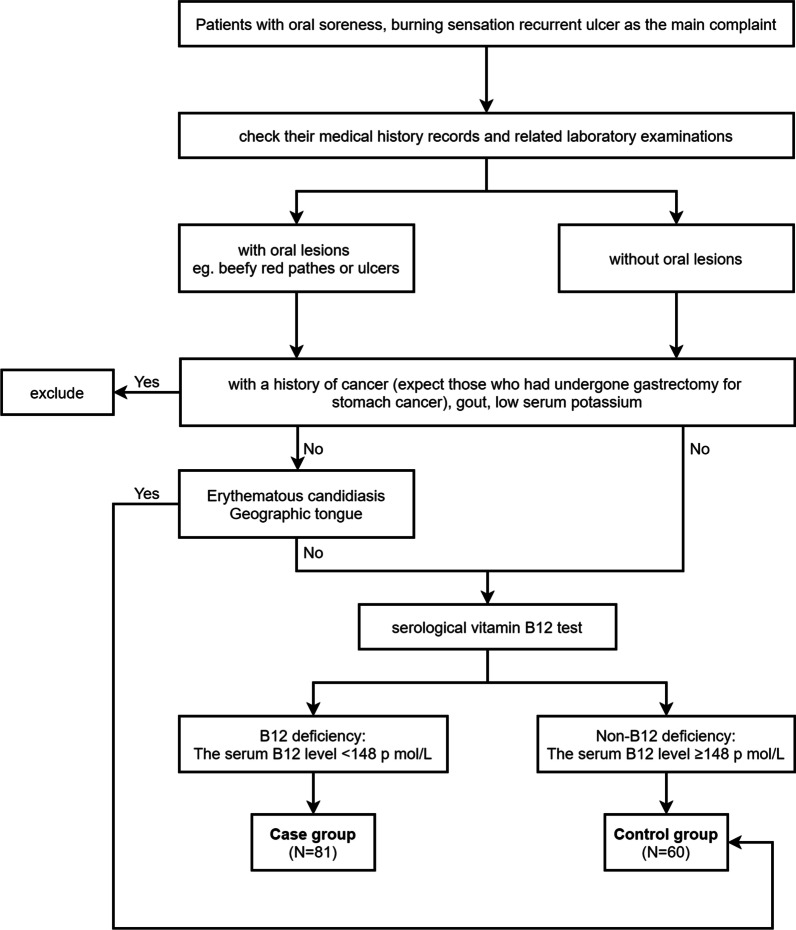
Schematic of grouping

The abundance of vitamin B12 in the serum is usually used to estimate the B12 status, and 148 pmol/L is established as the threshold for the diagnosis of the VB12 dosage [23]. According to the serum VB12 test results, the patients were divided into the case group (serum VB12 level < 148 pmol/L) and the control group (serum VB12 level ≥ 148 pmol/L) (Table 1). The whole grouping process is described in Fig. 1.

**Table 1:** Grouping standard

Group	Case group	Control group
Standard	Serum VB12 level < 148 pmol/L	
	VB12 deficiency: Serum VB12 level < 148 pmol/L	Non-VB12 deficiency: Serum VB12 level ≥ 148 pmol/L

VB12: Vitamin B12

### Statistical analysis

Parametric and nonparametric statistical tests were used for data analysis. When continuous variables conform to a normal distribution, an independent t-test was used when comparing the means between groups, while the frequency or percentage difference in the enumeration data was compared using the chi-square test.

Receiver operating characteristic curve (ROC) is used as a cutoff in diagnostic experiments. In this research, the sensitivity, specificity, Youden index (evaluates the validity of a diagnostic test), Kappa value, and area under the curve (AUC, evaluates the diagnostic efficiency of a diagnostic test) were statistically analyzed to assess the diagnostic efficiency and accuracy of different indicators, with the serological vitamin B12 as the gold standard diagnosis method.

Statistical analysis was performed using SPSS 20.0 (SPSS Inc., Chicago, IL, USA). *P* < 0.05 indicated statistical significance.

## Results

### Age and gender distribution

The present study included 141 patients: 81 in the case group and 60 in the control group. The age of the patients in the case and control groups was 60.07 ± 13.11 and 57.00 ± 12.30 years old, respectively, albeit no significant difference was observed. Also, the sex ratio did not differ significantly (P > 0.05) (Table 2).

**Table 2:** Clinical characteristics of participants

	Case group (n = 81)	Control group (n = 60)	*P* value
Age (mean ± SD, years)	60.07 ± 13.110	57.00 ± 12.295	> 0.05
Gender (male/female)	27/54	15/45	> 0.05
Beefy red patch	78	7	< 0.001
Oral ulcer	29	6	< 0.01
Stomach disease	13	2	< 0.05
MCV > 100 fL	68	4	< 0.001
Folic acid < 15.9 nmol/L	5	0	< 0.05

MCV: mean corpuscular volume

### Oral lesions

**Fig. 2:**
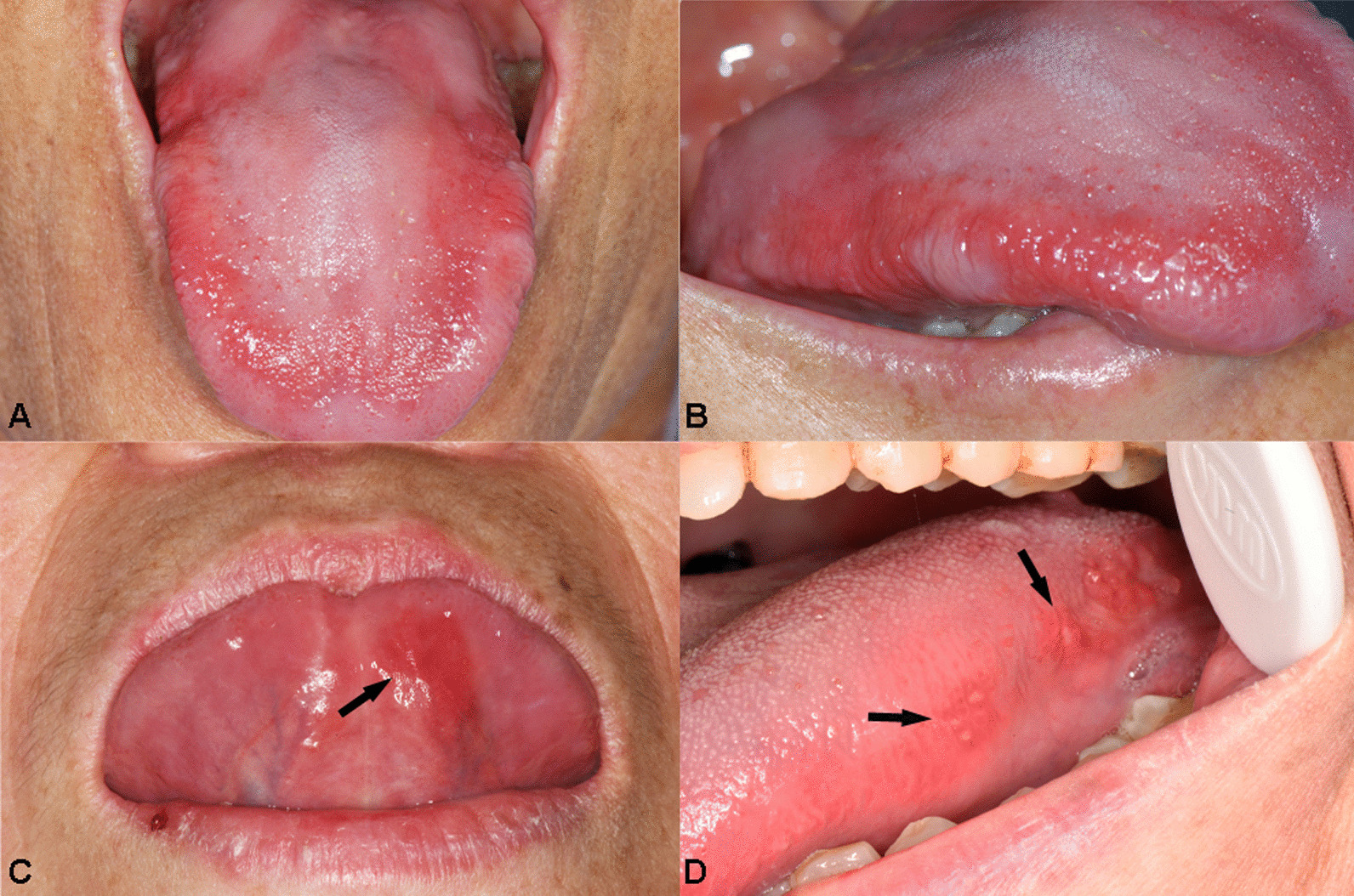
Different oral manifestations of VB12 deficiency. **a**, **b** Extensive linear or band-like “beefy red” patches on the lingual dorsum and lingual marginal in VB12 deficiency. **c** Localized “beefy red” patches on the underside of the tongue. **d** Ulcer on the ventral region of the tongue

Oral mucosal “beefy red” patch refers to recurring mucosal pain, atrophy of tongue papilla, irregular congestion, and redness (Fig. 2A, B). In the case group, patients showed irregular “beefy red” patch on the cheeks, back of the tongue, the belly of the tongue, and floor of the mouth. In some cases, the atrophy of the tongue papilla and frequent ulcers were also detected. The ulcer could be located on the tongue, the belly of the tongue, posterior part of the soft palate, about 1–2 mm in size, and obvious pain, and easily misdiagnosed as ordinary RAU (Fig. 2C, D). The proportion of “beefy red” patch and oral ulcers in the case group was significantly higher than that in the control group. In the case group, the patients had more “beefy red” patch than oral ulcers (Table 2).

### Systemic symptoms and stomach diseases

The onset of VB12 deficiency is hidden, and subclinical symptoms, such as fatigue, weakness, and anorexia may appear in the early stage. As the disease progresses, megaloblastic anemia, skin and mucous membrane pigmentation, mental depression, fantasy, dementia, and other irreversible mental damages appear [24]. In this study, few patients experienced fatigue, but none had psychiatric symptoms or injuries.

VB12 deficiency affects the DNA synthesis of mucosal cells in the digestive tract. When the function of the digestive tract is impaired, a series of gastrointestinal disorders, such as anorexia, indigestion, bloating, and diarrhea occur [2, 24]. The investigation of the medical history revealed that 13 individuals in the case group were known to have the gastric disease, while there were 2 people with the gastric disease in the control group. The difference between the two groups was also statistically significant (*P* < 0.05) (Table 2).

### Results of clinical and hematological examinations

VB12 deficiency can be manifested as megaloblastic anemia, which could be detected by blood routine examination. When B12 is deficient, methyl cannot be transferred, and tetrahydrofolate cannot be regenerated, which affects DNA synthesis and slows down cell proliferation. However, RNA accumulates, the red blood cell body is enlarged, the nucleoplasm is developed and separated, and megaloblasts are formed. The lifespan of the red blood cells is shortened, and early hemolysis is ineffective hematopoiesis. A blood routine was performed in both groups. In the case group, the number of patients with MCV > 100 fL reached 68, while only 4 were noted in the control group. The difference between the two groups was statistically significant (*P* < 0.001). VB12 deficiency is associated with an increase in MCV. Only 5 patients were noted with abnormal serum folic acid in the case group and none in the control group (*P* < 0.05). These findings suggested a correlation between VB12 deficiency and a decrease in folate content.

### Diagnostic test

In order to elucidate the diagnostic potential of oral manifestations and routine serological testing results for the disease, an appropriate test was carried out. Herein, we analyzed the diagnostic value of the oral lesions (“beefy red” patch) and MCV (80–100 fL) for VB12 deficiency (Table 3).

**Table 3:** Value of indices diagnosis of B12 deficiency

	N	Validity			Reliability	Accuracy
		Sensitivity	Specificity	Youden index*	Kappa value	AUC
Beefy red patch	78	0.963	0.883	0.846	0.853	0.923
MCV > 100 fL	68	0.815	0.933	0.748	0.758	0.920
MCV > 100 fL and “beefy red” patch	65	0.840	0.950	0.790	0.761	0.949

MCV: mean corpuscular volume

*Youden index = Sensitivity + Specificity-1

Based on the perspective of a single index, the sensitivity of “beefy red” patch was > 0.9, and the specificity was > 0.8, while the sensitivity of MCV was > 0.8, and the specificity was > 0.9. The specificity of MCV was higher than that of the other index (mucosal “beefy red” patch); thus, the Youden index of mucosal “beefy red” patch and the ability to correctly diagnose B12 deficiency were higher, and the Kappa value and the AUC of “beefy red” patch were greater than that of MCV, indicating the diagnostic effect of “beefy red” patch was greater than MCV.

The diagnostic value of MCV combined with oral manifestations was analyzed further. The specificity (0.950) of “MCV > 100 fL” combined with “beefy red” patch are higher than that of MCV or “beefy red” patch. Compared to a single index, the AUC (0.949) of “beefy red” patch was highest, but the Kappa value of “MCV > 100 fL and beefy red” patch was below that of “beefy red” patch. Typically, the specificity and AUC of “MCV > 100 fL” and “beefy red” patch were highest, which means lower misdiagnosis rate and higher diagnostic accuracy.

## Discussion

Clinically, the medical environment of primary hospitals is poor, and expensive serological tests cannot be popularized, which might delay the timely treatment of the disease. Therefore, this study evaluated the diagnostic efficiency of fingertip blood MCV and oral lesions for VB12 deficiency, which are simple, time-saving, easy, and suitable for local clinics and hospitals without in-house clinical laboratories, especially the remote areas.

In this study, the average age of the patients in the two groups was > 55-year-old, and the cohort consisted of more women than men. The patients in the case group showed oral lesions and/or MCV abnormalities. The most common positive test result was “beefy red” patch, followed by MCV > 100 fL, indicating that oral mucosal “beefy red” patch is common in patients with VB12 deficiency. Previous studies proposed that the accelerated metabolism of oral mucosal cells cause oral lesions to manifest as oral mucosal “beefy red” patch [25]. Therefore, we concluded that the appearance of oral mucosal irregular red patches might be a characteristic manifestation of VB12 deficiency. Its diagnostic value showed a satisfactory sensitivity (0.963) and specificity (0.833); however, the red patches on the tongue due to *Candida* infection need to be ruled out. Only the MCV value has low specificity for diagnosing VB12 or folate deficiency [26, 27]. Although MCV is within the normal range of 80–100 fL, using it as a standard to diagnose patients with VB12 deficiency has only a 25% chance of success [28, 29]. In the present study, the abnormal MCV of VB12 deficiency is obvious. However, the diagnosis efficiency of MCV is not high and could be missed easily. The sensitivity, specificity, and diagnostic accuracy of MCV combined with oral lesions are improved. In the case of serum-free testing, the diagnostic treatment of VB12 supplementation could be carried out according to the result of routine serological testing with minor trauma and clinically visible oral mucosal lesions.

In the process of diagnosing VB12 deficiency, we have key points that need to be emphasized. Methylcobalamin is the form of VB12 coenzyme that participates in the metabolism of folic acid and the production of tetrahydrofolate and promotes the turnover and utilization of folic acid. The lack of VB12 affects the production and especially the utilization of folic acid [2, 30, 31]. In this study, only 5/81 (6.17%) patients showed folic acid deficiency in the case group, indicating that VB12 deficiency appears earlier than the reduction of folic acid. In most early stages, patients with VB12 deficiency with “beefy red” patch in the oral mucosa and the main complaint of pain, the folic acid concentration is in the normal range but may have subtle effects on the nervous system that are easily ignored [2]. Thus, when VB12 deficiency is diagnosed, folic acid should be supplemented appropriately while supplementing VB12. In addition, the results of the present study showed that of the 35 oral ulcer patients, 29 had VB12 deficiency. Therefore, we speculated that when the patient had a beefy red patch on the oral mucosa with oral ulcers or when anemia/megaloblastic anemia was detected in the routine blood examination or when oral mucosal ulcers occur, and the conventional treatment methods for RAU are not effective, the patients should be cautious of the possibility of VB12 deficiency.

6–30% of the elderly suffer from VB12 deficiency, and the prevalence increases with age [26, 32, 33]. Laboratory tests of elderly >65-years-old demonstrated that 63/1048 (6.1%) had low serum total VB12, but only 27/1048 (2.6%) subjects had previously diagnosed VB12 deficiency [34]. VB12 deficiency often occurs in areas of poverty in rural areas [35], where these conditions make the diagnosis of VB12 deficiency difficult. The present study provides a simple and feasible method for the diagnosis of VB12 deficiency in the elderly in local clinics and hospitals without in-house clinical laboratories in underdeveloped countries or remote areas. Considering that timely diagnosis and appropriate treatment prevent VB12 deficiency-related complications, future studies are needed to verify the effectiveness of the current method using a large sample size.

## Conclusions

MCV > 100 fL combined with oral mucosal “beefy red” patch could be conducive to early diagnosis in local clinics and hospitals without in-house clinical laboratories, which is used for the diagnostic treatment with VB12 supplementation. If patients with oral ulcers do not perform satisfactorily according to conventional RAU treatment, especially elderly patients or patients with gastrointestinal diseases, it might further examination to rule out VB12 deficiency.

## Data Availability

The datasets used/or analyzed during the current study are available from the corresponding author on reasonable request.

## Abbreviations

VB12: Vitamin B12
MCV: Mean corpuscular volume
RAU: Recurrent aphthous ulcer
ROC: Receiver operating characteristic curve
AUC: Area under the curve
